# Genetic Diversity and Differentiation of Eleven *Medicago* Species from Campania Region Revealed by Nuclear and Chloroplast Microsatellites Markers

**DOI:** 10.3390/genes13010097

**Published:** 2021-12-31

**Authors:** Imene Khadidja Djedid, Mattia Terzaghi, Giuseppe Brundu, Angela Cicatelli, Meriem Laouar, Francesco Guarino, Stefano Castiglione

**Affiliations:** 1 Laboratory of Integrative Improvement of Vegetal Productions, Higher National Agronomic School (ENSA), Algiers DZ16200, Algeria; i.djedid@edu.ensa.dz (I.K.D.); m.laouar@ensa.dz (M.L.); 2 Department of Chemistry and Biology “A. Zambelli”, University of Salerno, 84084 Fisciano, Italy; mterzaghi@unisa.it (M.T.); acicatelli@unisa.it (A.C.); scastiglione@unisa.it (S.C.); 3 Department of Agricultural Sciences, University of Sassari, Viale Italia 39, 07100 Sassari, Italy; gbrundu@uniss.it

**Keywords:** SSR markers, universal chloroplast SSRs, *Medicago*, genetic analyses, nuclear microsatellite

## Abstract

The species belonging to the genus *Medicago* are considered a very important genetic resource at global level both for planet’s food security and for sustainable rangelands management. The checklist of the Italian flora (2021) includes a total number of 40 *Medicago* species for Italy, and 27 for Campania region, with a number of doubtful records or related to species no more found in the wild. In this study, 10 *Medicago* species native to Campania region, and one archaeophyte (*M. sativa)*, identified by means of morphological diagnostic characters, were analyzed in a blind test to assay the efficacy of nine microsatellite markers (five cp-SSRs and four n-SSRs). A total number of 33 individuals from 6 locations were sampled and genotyped. All markers were polymorphic, 40 alleles were obtained with n-SSRs ranging from 8–12 alleles per locus with an average of 10 alleles per marker, PIC values ranged from 0.672 to 0.847, and the most polymorphic SSR was MTIC 564. The cp-SSRs markers were highly polymorphic too; PIC values ranged from 0.644 to 0.891 with an average of 0.776, the most polymorphic cp-SSR was CCMP10. 56 alleles were obtained with cp-SSRs ranging from 7 to 17 alleles per locus with an average of 11. AMOVA analysis with n-SSR markers highlighted a great level of genetic differentiation among the 11 species, with a statistically significant fixation index (*F*_ST_). UPGMA clustering and Bayesian-based population structure analysis assigned these 11 species to two main clusters, but the distribution of species within clusters was not the same for the two analyses. In conclusion, our results demonstrated that the combination of the used SSRs well distinguished the 11 *Medicago* species. Moreover, our results demonstrated that the use of a limited number of SSRs might be considered for further genetic studies on other *Medicago* species.

## 1. Introduction

Fabaceae, the third-largest Angiosperm family with 751 genera and 19,400 species [1], is a highly diversified plant family and features many economically important crops, ranging from food crops to fodder species. It provides about one-third of protein for human consumption and a wide range of raw materials for industries [2]. Legumes are able to improve soil fertility by fixing atmospheric nitrogen through symbiotic bacteria (*Rhizobium*) and have an important role in the global nitrogen cycle [3,4,5,6].

Among the six legumes subfamilies, Papilionoideae is the richest for number of species and the most studied one, embodying 476 genera and 13,860 species [2,7,8,9,10,11]. In this subfamily, the genus *Medicago* is arguably one of the most common in Mediterranean and warm-temperate grassland and shrubland, featuring 14 sections and 87 species, including annual and perennial species [4,12,13]. It shows a great species diversity and excellent prospects for breeding and to enhance provision of high quality winter fodder resources. In the Mediterranean basin, *Medicago* species can be used as useful pioneer plants for the improvement of marginal land [14,15]. In addition to producing good quality fodder [16], *Medicago* annual species are self-sowing and therefore very valuable in crop rotations with cereals, in low input arid farming systems. Moreover, a number of *Medicago* species have potential for the production of bio-pharmaceuticals, -fuel, and -plastics [4,17].

Since 1753, Carl von Linné [18] described 19 species of the genus *Medicago.* A first attempt at synthesis was made by Urban in 1872 [19], but in a relatively incomplete manner. In 1963, Heyn studied this genus detailing particularly the taxa of the *Spirocarpos* section [20] and producing a monograph on the genus listing 54 species belonging to 4 subgenera. The most important and recent enrichment of the genus arises particularly from the inclusion of 23 *Trigonella* species [21]. 

Currently, out of 87 *Medicago* species, 21 are perennial [4,22]. In fact, the most supported hypothesis suggests that the ancestral state of the genus *Medicago* is perennial allogamous, and that the annual self-pollinated species are derived from those progenitors [4,23,24]. The annual species are confined particularly to the Mediterranean basin, while the perennial species are more widely distributed, and are rather located in the East of the Mediterranean and in Central and Western Asia.

Common and rare species within the genus *Medicago* have been reported from different countries of the Mediterranean basin [25], being a number of species included in the European Red List of the vascular plants [26]. No comprehensive synopsis on the systematics of this group is available for Italy [27], however, in the checklist of the Italian Flora (2021), a total number of 40 *Medicago* species is reported, although a number of these records are doubtful or related to species no more present in the wild. According to the same source 27 *Medicago* species are present in the Campania region. In Sardinia, *M. polymorpha* and *M. arabica* are the most abundant followed by *M. murex*, *M. truncatula*, *M. orbicularis*, and *M. minima* [28]. In Sicily, *M. polymorpha* and *M. orbicularis* are very common and the large pod species (*M. ciliaris*, *M. intertexta,* and *M. scutellata*) are more frequent in non-grazed cultivated areas, while other species (*M. murex*, *M. truncatula*, *M. arabica*, *M. minima,* and *M. rigidula*) are more frequent in rangelands [29]. Among perennial species, *Medicago falcata* subsp. *falcata* and *M. marina* are native to Italy, the latter being particularly threatened by trampling and habitat modification in sand dune systems.

In addition to morphological identification and study of diagnostic traits, molecular tools can help to solve a number of taxonomic ambiguities [30]. For example, microsatellites are very valuable molecular tools in genetic diversity assessment and linkage mapping. In most Angiosperm, n-SSR markers are highly reproducible, phylogenetically significant, and with biparental inheritance, hence highly recommended for the evaluation of contemporary patterns of genetic structures. Microsatellites are available in the model plant *M. truncatula* [31,32]. The high level of synteny between legume genomes allowed the transferability of SSR markers developed on the genome of *M. truncatula* [33] (more than 500 SSR) to the other *Medicago* species [21,34,35,36]. Contrariwise, chloroplast genomes have lower evolutionary rate, are predominantly not recombining, maternally inherited, highly conserved across genera, and often distributed throughout noncoding regions. Polymorphic mononucleotide repeats (cp-SSR) exhibit length variation in the number of repeats akin to nuclear SSR (n-SSR), so they are powerful markers for genetic studies of ancient historical relationships. The combined analysis of biparentally and maternally inherited microsatellite markers would be expected to provide suitable complementary or sometime discordant information on the genetic diversity, structure, and differentiation of *Medicago* species [37,38,39,40,41,42].

The present study is a first attempt aiming to assess whether the use of a limited set of nuclear and/or chloroplast microsatellites is an effective tool for *Medicago* species and taxonomic sections’ differentiation, and complementary to morphological identification. 

## 2. Materials and Method 

### 2.1. Plant Material

**Figure 1:**
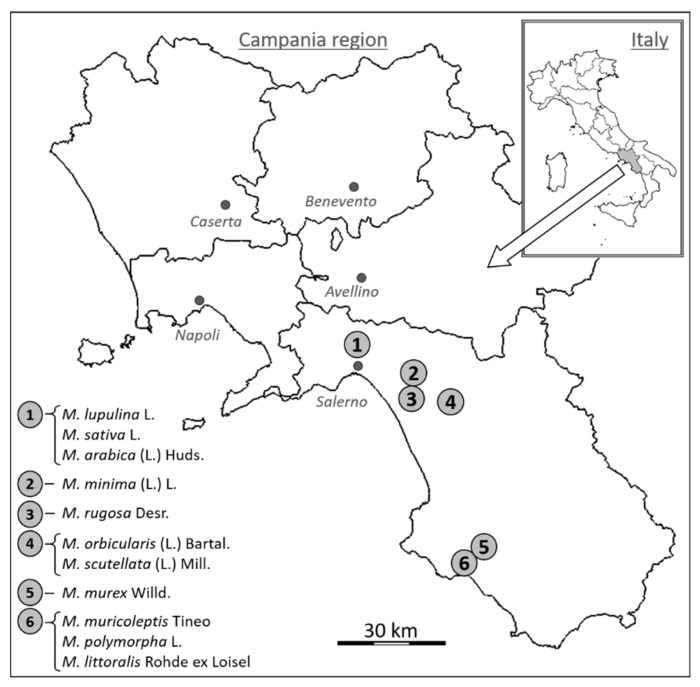
Map of Campania region, in southern Italy, showing the locations of the six collection sites of the eleven *Medicago* species investigated.

During field surveys and laboratory observations, 11 *Medicago* species were identified based on their morphological characters of vegetative and reproductive organs. We considered a large number of characters, such as, e.g., petiole length, leaflet margin, stipule length, stipule margin, stipule shape, inflorescence length, number of flowers per inflorescence, pod length, pod width, etc. These 11 *Medicago* species included one perennial, one biannual, or short-lived perennial, and nine annual species. Aiming to analyze 3 individuals for each species (Table 1), 33 specimens were collected along secondary or dust road verges in the Province of Salerno (Campania region, Southern Italy, latitude 40.07–40.77°, longitude 14.78–15.55°). All collection sites were GPS georeferenced and plotted on a geographic map (Table 1, Figure 1). 

### 2.2. DNA Extraction and SSR Analysis

For each Medicago species, leaf samples were harvested from three individuals representative of the population thriving in the collection site, and then stored in silica-gel before DNA extraction and purification. Leaf samples were randomly numbered to perform a blind test. Afterwards, leaves were powdered in liquid nitrogen and total DNA was extracted using a cetyl-trimethyl-ammonium bromide (CTAB) buffer, following the protocol of Doyle and Doyle (1987), as modified by Harbor, Doyle and Tai [43,44,45]. DNA quantity and quality were assessed using 1.0% agarose gel electrophoresis. Genetic analysis was carried out by means of: (a) four nuclear microsatellites (n-SSR) from *M. truncatula* selected on the basis of their position on the genetic linkage map [46] (MTIC 503, MTIC 559, MTIC 563, MTIC 564) (Table S1); (b) five chloroplast Simple Sequence Repeat (cp-SSR) markers comprising CCMP2, CCMP4, CCMP6, CCMP7, and CCMP10, designed for *Nicotiana tabacum* L. [47,48] (Table S2).

### 2.3. N-SSR and Genetic Diversity Analysis

PCR amplifications were performed using a thermocycler (2720 thermocycler, Applied Biosystems, Italy) in 10.0 μL of a mixture solution containing 10 ng of *Medicago* template DNA, 2.0 μL of reverse primer (final concentration 1.0 μM, unlabeled), 1.5 μL of forward primer (final concentration 1.0 μM, unlabeled), 0.5 μL of labelled forward primer (final concentration 0.1 μM, unlabeled), and 5.0 μL of RedExtract-N-Amp PCR Ready Mix (Sigma-Aldrich, Milano, Italy), following a Touchdown PCR thermal profile of 4 min at 94 °C, 15 cycles of 30 s at 94 °C, 30 s at 62 °C with −0.5 °C decrease at each cycle plus 1.0 min at 72 °C and 30 cycles of 30 s at 94 °C, 1 min at 54 °C plus 1 min 30 s at 72 °C, succeeded by 7 min at 72 °C. PCR products were separated by agarose gel electrophoresis 1.0% in 1× TAE buffer, stained with ethidium bromide and visualized under UV light. For electrophoresis capillary, an aliquot of 1.0 µL of the PCR amplicons was diluted in 10 μL of formamide and 0.5 μL of Rox 500™ oligonucleotide ‘size ladder’ (MCLAB, South San Francisco, CA, USA) then analyzed in ABI-PRISM 310 Genetic Analyzer (Applied Biosystems^®^, Monza, Italy). Alleles sizes were scored using Gene Mapper 4.0 (Applied Biosystems^®^, Monza, Italy).

#### 2.3.1. Genetic Diversity Analysis

Gene diversity and allele number (A) for each locus were obtained using Molecular kinships ver. 3.0. software, PIC values were calculated using the following formula for molecular markers [49]:

PIC*_i_* = 2·f*_i_*·(1 − f*_i_*) (1)

where PIC*_i_* is the polymorphism information content of the *i* allele and f*_i_* is the frequency of amplification (presence of fragment) of the *i* allele in the analyzed individuals.

In order to estimate the level of genetic diversity present in the species from n-SSR data, banding profiles generated by each marker were scored on the basis of the size (bp) of amplified fragments. Deviation from Hardy–Weinberg Equilibrium (HWE) was tested at both species and locus levels and inbreeding coefficients (*F*) were calculated using GENEPOP software (ver. 4.7.5, Montpellier, France). 

The following genetic diversity indices were calculated using GenAlEx software (ver. 6.503) [50]:

(i) The total number of alleles (*Na*);

(ii) The effective number of alleles (*Ne*);

(iii) Observed heterozygosity (*H*_o_);

(iv) Expected heterozygosity (*H_E_*);

(v) Shannon’s information index (*I*);

(vi) Gene flow (Nm) was calculated using the following formula [50]:

Nm = (1 − *F*_ST_) / 4*F*_ST_ (2)

In order to assess the variance among and within species and to estimate genetic differentiation among *Medicago* species, analysis of molecular variance (AMOVA) was performed using 1000 permutations of the *F*_ST_ value; principal coordinates analysis (PCoA) and relationships between genetic and linear geographic distances (isolation by distances, IBD), were examined using a Mantel test [51] as implemented in GenAIEx, with 1000 permutations. 

The alleles banding profiles were transformed into a binary matrix of presence (1)/absence (0) of each allele and genetic relationships were visualized using cluster analysis and the R package ‘pvclust’ [52], based on Euclidean distance, since this method has been proved to be the most appropriate for recognizing the genetic structure extractable from the analyzed dataset.

#### 2.3.2. Genetic Structure Analysis

The genetic structure was investigated with a Bayesian approach with Structure software (ver. 2.3.4) [53], through 100,000 Monte Carlo Markov Chain (MCMC) iterations, following 25,000 burn-in length for each run. Eleven independent simulations and eight replicates were conducted for each K-value to estimate group assignments.

The analyses were conducted combining two different models (admixture/no-admixture) and two options of allele frequencies among species (correlated/independent), and the other parameters were set to default values as suggested by Pritchard et al. [54]. Structure Harvester b (v0.6.94) [55] was used to select the optimal model relying on maximum likelihood and (ΔK) values. ΔK based on the order rate of change of L(K) between successive K values, was used to identify the correct number of K [56].

### 2.4. Cp-SSRs and Statistical Analysis

PCR reactions were performed in 10 μL reaction mixture containing 10 ng of template DNA, 1.5 μL of forward primer (final concentration 1.0 μM, unlabeled) 0.5 μL of labeled forward primer (final concentration 0.1 μM), 2.0 μL of reverse unlabeled primer (final concentration 1.0 μM, unlabeled), 5 μL of RedExtract-N-Amp PCR Ready Mix (Sigma-Aldrich, Milan, Italy) with an initial melting temperature set at 94 °C and maintained for 3 min; 15 cycles of 94 °C for 30 s, 1 min at the annealing temperature of 62 °C with −1 °C decrease at each cycle and 25 cycles at the denaturation temperature of 94 °C for 30 s, 55 °C for 1 min, extension at 72 °C for 1 min 30 s, and then a final extension step at 72 °C for 7 min. The amplified PCR products were resolved on 1.0% agarose gel electrophoresis and visualized by ethidium bromide staining then analyzed by capillary electrophoresis with an automatic sequencer, aiming at characterizing allelic diversity and polymorphic informativeness of cp-SSRs of our *Medicago* species, PIC values and alleles number (A) were calculated using Molecular Kinships software (ver. 3.0), and the gene diversity (*H_e_*) was calculated as follows:

(3) $$H_{e}=1-\sum_{i=n}^{}\;pi^{2}$$

where *n* is the number of alleles and *p_i_* the frequency of the *i*th allele in species. Haplotypes Diversity was calculated in the same manner with n and *p_i_* referring to haplotypes.

An unweighted pair group method with arithmetic mean (UPGMA) clustering analysis was run with NTSYS pc 2.02j software [57], using a clustering algorithm based on the Jaccard similarity index [58]. The Reliability of SSR allele clustering was assessed by bootstrapping, with 1000 permutations.

## 3. Results

### 3.1. Genetic Diversity at Nuclear Microsatellites

The four n-SSR were found to be highly polymorphic, with allele number per locus ranging from 8 (MTIC 559) to 12 (MTIC 564), as reported in Table 2. A total of 40 alleles were amplified from the DNA of the 11 *Medicago* species, with a mean value of 10 alleles per SSR locus. Gene diversity (*He*) per locus ranged from 0.695 (MTIC 563) to 0.861 (MTIC 503) with an average of 0.799. The Polymorphism Information Content (PIC) for each primer was in the range of 0.672 to 0.847 with an average of 0.780.

The results of n-SSR (Table 2) suggested a moderate level of genetic diversity of the studied *Medicago* species: Na values ranged from 1.2 (POL) to 2.2 (SAT and SCU), Ne values from 1.2 (POL) to 2.2 (SCU and ORB), HO and He values ranged from 0.00 (POL) to 0.33 (SAT) and from 0.11 (POL) to 0.46 (ARA), respectively. Within the 11 species, the average number of alleles revealed by the surveyed loci was 6.3; it ranged from 3 (POL) to 8 (SAT, ARA, LIT and MRX). Although the number of migrants (Nm) ranged from −0.64 (LUP) to 0.49 (RUG).

Inbreeding indices (F) deviated from zero for almost all the species, they were in the range of −0.33 (LUP) to 1.00 (POL) and showed heterozygote deficiencies: a total loss of heterozygosity in *M. polymorpha* and a loss of 33% of homozygosity in *M. lupulina* (Table 3).

The results of the overall AMOVA (Table 4) indicated that 52% of the variation was due to differences among species, while the remaining 48% was due to the variation within groups.

**Figure 2:**
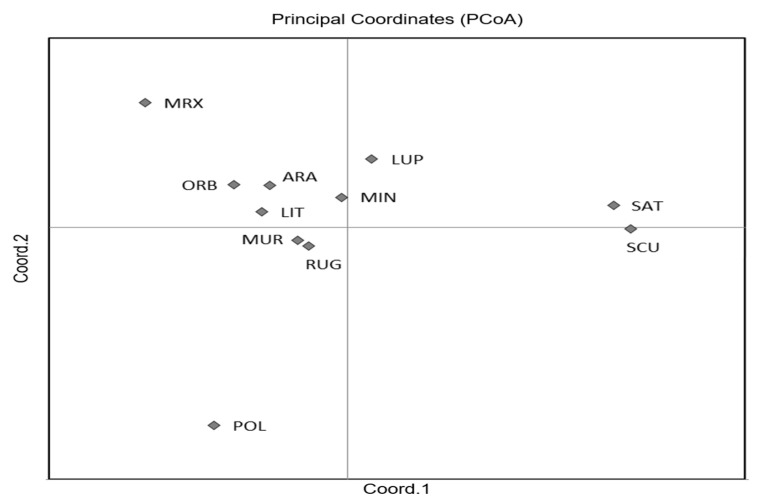
Plot of Principal Coordinate Analysis (PCoA) for the 11 *Medicago* species. Codes are as follows: SCU, *M. scutellata*; SAT, *M. sativa*; LUP, *M. lupulina*; ARA, *M. arabica*; MUR, *M. muricoleptis* POL, *M. polymorpha*; ORB, *M. orbicularis*; MIN, *M. minima*; LIT, *M. littoralis*; RUG, *M. rugosa*; MRX, *M. murex*.

The first two axes of the PCoA (Figure 2) explained 70.65% of the total variation and clearly separated *M. sativa* and *M. scutellata* from all the other species, with an accumulated variance of 42.21% and 28.44%, respectively. Although the *F*_ST_ values ranged from 0.03 to 0.333 (Table S3), indicating a moderate or great genetic differentiation, the two highest differentiations were observed between *M. scutellata* and *M. murex*, and *M. scutellata* and *M. polymorpha*, whilst the lowest differentiation was found between *M. minima* and *M. rugosa*.

**Figure 3:**
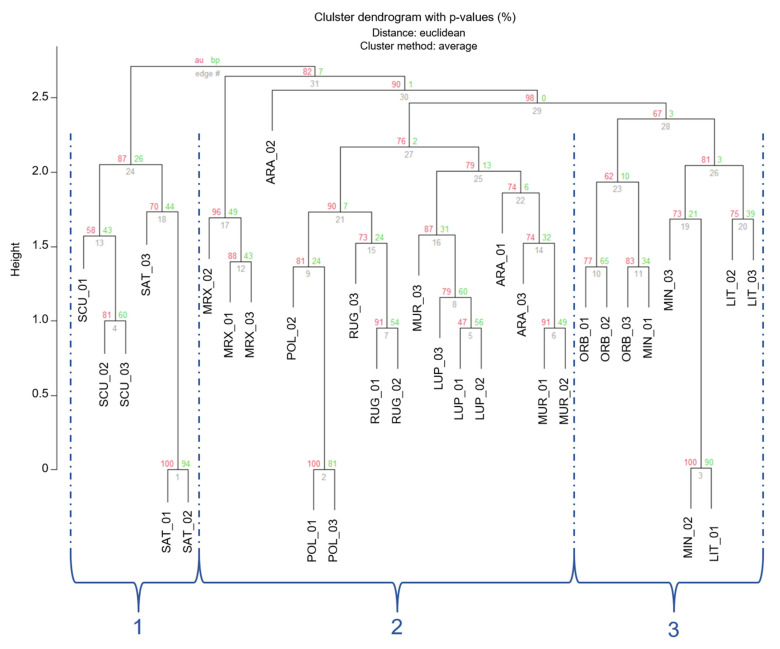
Cluster dendrogram of the 33 *Medicago* individuals based on the Euclidean distances. Values at branches are AU *p*-values (red) and Bootstraps BP-values (green), based on n-SSR primers. Codes are as follows: SCU, *M. scutellata*; SAT, *M. sativa*; LUP, *M. lupulina*; ARA, *M. arabica*; MUR, *M. muricoleptis* POL, *M. polymorpha*; ORB, *M. orbicularis*; MIN, *M. minima*; LIT, *M. littoralis*; RUG, *M. rugosa*; MRX, *M. murex*.

The clustering, based on Euclidean distances (Figure 3), showed a partition of individuals in three main groups (1–3 in Figure 3), which were separated by an Euclidean distance value of 2.75 and 2.56, respectively. In order to read these results and for a better approximation to an unbiased *p*-value (AU), AU values were adopted instead of BP-values [59]. Nonetheless, approximately AU values were not significant (AU < 95) for most branches. However, individuals belonging to the 3 different *Medicago* sections were located in different clusters.

The first smaller branch of cluster 1 included individuals from *Medicago* and *Spirocarpos* subsection *Rotatae*; the cluster 2 included individuals belonging to *Orbiculares* and to two *Spirocarpos* subsections (*Pachyspirae* and *Spirocarpos*). All the others grouped in cluster 3 and included species belonging to *Spirocarpos* section and a number of subsections (e.g., *Rotatae*, *Lupularia,* etc.).

**Figure 4:**
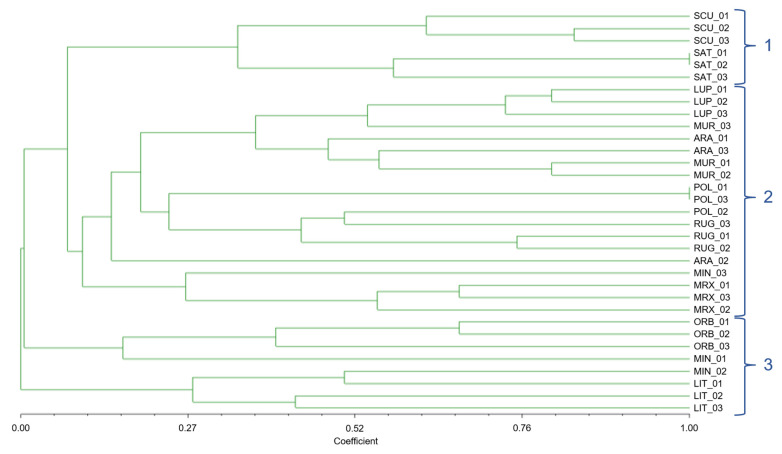
Dendrogram based on Jaccard similarity indices, computed on the basis of UPGMA clustering method, of the 33 individuals of 11 *Medicago* species, belonging to 3 different sections, with n-SSR alleles.

The 33 individuals of 11 species were divided into almost the same groups by the UPGMA dendrogram based on the Jaccard similarity index as by the Pvclust clustering method; although well distinguished (Figure 4), *M. scutellata* and *M. sativa* formed a separate cluster (group 1); *M. littoralis* and *M. minima* (section *Spirocarpos*) formed the cluster number 3 together with *M. orbicularis* (section *Orbiculares*). In contrast, all other investigated species were grouped in cluster 2.

### 3.2. Genetic Structure at Nuclear Microsatellites

**Figure 5:**
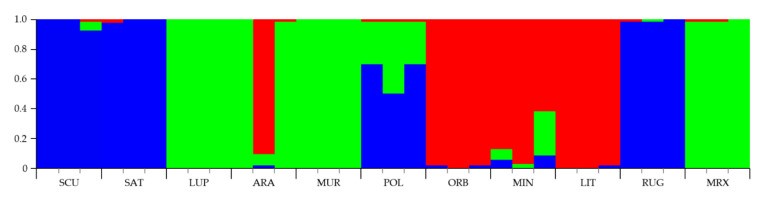
Bar plot of genetic relationships among the 11 *Medicago* species estimated using STRUCTURE software based on the data obtained by the four n-SSR loci analyzed. Codes are as follows: SCU, *M. scutellata*; SAT, *M. sativa*; LUP, *M. lupulina*; ARA, *M. arabica*; MUR, *M. muricoleptis;* POL, *M. polymorpha*; ORB, *M. orbicularis*; MIN, *M. minima*; LIT, *M. littoralis*; RUG, *M. rugosa*; MRX, *M. murex*. The estimated membership probability (Q) for K = 3 is plotted on the y-axis.

To assign individuals to one or more estimated groups (K), a Bayesian Markov Chain Monte Carlo approach, implemented in Structure (ver. 2.3) [53], was applied under the no-admixture model and with the assumption of independent allele frequencies between species. At K = 2, *Medicago* species were assigned into groups belonging to different sections. At K = 3 (Figure 5), species belonging to the 3 sections were grouped in different subgroups: *M. sativa*, *M. scutellata*, and *M. rugosa* were allocated to one subgroup (cluster 1, blue); *M. lupulina*, *M. arabica*, *M. muricoleptis*, and *M. murex* in cluster 2 (green), while the cluster 3 (red) was formed by *M. littoralis*, *M. minima*, and *M. orbicularis*. It is worthwhile to note that *M. polymorpha* had the highest average ancestry coefficient (inferred proportion of membership) from the cluster 1 (0.7) then from cluster 2 (0.3). Consequently, K = 3 was recognized as the most appropriate value able to describe the genetic structure of the 11 *Medicago* species studied (Figure 5). 

### 3.3. Genetic Analyses by Chloroplast Microsatellites

The statistics results for the five cp-SSR markers are summarized in Table 2. The loci showed a high level of genetic diversity. In total, 56 polymorphic alleles were amplified, ranging from 7 alleles per locus in the case of CCMP6, to 17 alleles per locus in the case of CCMP10, with an average of 11. Gene diversity (*He*) per locus ranged from 0.694 (CCMP6) to 0.899 (CCMP10), with an average of 0.833. Meanwhile, Polymorphism Information Content (PIC) of each primer was in the range of 0.694 (CCMP6) to 0.899 (CCMP10), with an average of 0.833. These results demonstrate that the cp-SSR markers used were enough informative to justify further *Medicago* species genetic diversity analysis. CCMP10 was the cp-SSR showing the highest ability to distinguish among the different analyzed species.

Gene diversity (*He*) for the 11 species (Table 3) varied from 0.09 (MRX) to 0.58 (MIN) with an average of 0.44. Allele numbers within species varied from 5 (MRX) to 10 (SCU, ARA, MIN).

The AMOVA (Table 4) showed that genetic variation was mainly within species (94%), rather than among species (6%). Although genetic differentiation among species was found from moderate to high, the PHI-PT values for haplotypes ranged from 0.083 to 0.600, the highest differentiation was observed between *M. orbicularis* and *M. murex.*

Mantel test for isolation by distance among species did not show any significant correlation between pairwise PHI-PT and geographic distance (R^2^ = 0.012, *p* = 0.158) [60].

**Figure 6:**
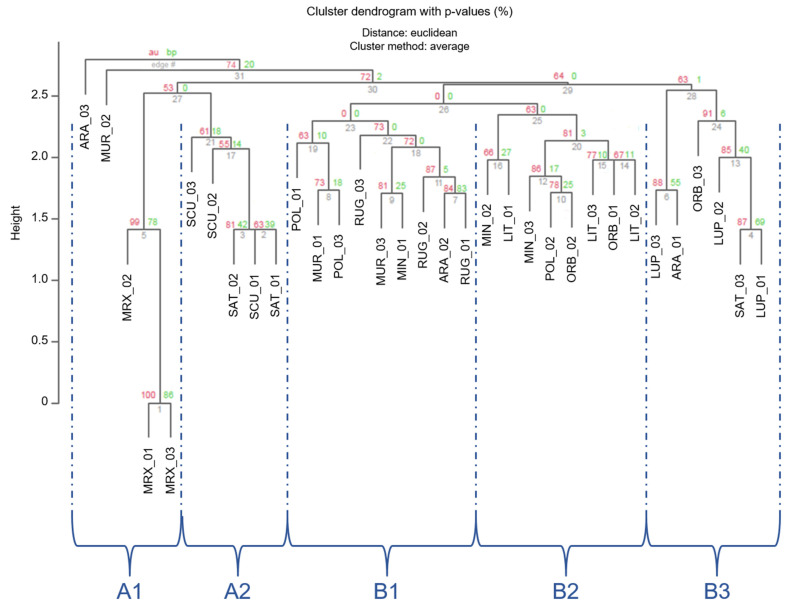
Cluster dendrogram of individuals based on the Euclidean distances. Values at branches are AU *p*-values (red) and Bootstraps BP-values (green), based on cp-SSRs.

Unbiased cluster analysis based on cp-SSR markers was performed with the Numerical Taxonomy Multivariate Analysis System (NTSYS-PC-ver. 2.2) (Figure 6) [3]. A dendrogram was built via UPGMA and Jaccard similarity coefficients were used to reveal the similarity among the 11 *Medicago* species. The results of clustering analysis with pvclust based on Euclidian distances revealed the presence of two macro-clusters (A and B). These two clusters included different sub-clusters, A1, A2, B1, B2, and B3. In particular, A1 was formed by the only *M. murex*, its specimens were well separated from *M. sativa* and *M. scutellata* cluster (A2). In the case of the macro cluster B, the three sub-clusters B1, B2, and B3 showed that different *Medicago* species grouped together, even if the three clusters were well separated. However, the identified subclusters comprised all the individuals of the same species (e.g., B1 grouped all the specimens belonging to *M. rugosa*, B2 all those belonging to *M. littoralis*, B3 the ones belonging to *M. lupulina*). In general, the AU value was quite well supported by data obtained, in particular in the case of the subgroups (AU comprised between 53% and 99%).

### 3.4. Genetic Similarity Analysis among Species Based on Chloroplast and Nuclear Microsatellites

#### 3.4.1. Pvclust-R-Package

**Figure 7:**
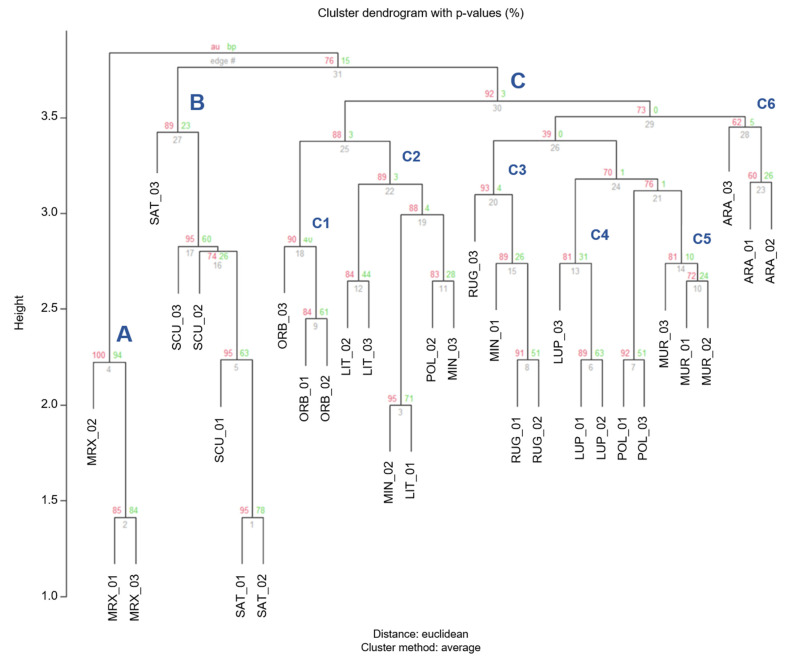
Cluster dendrogram of individuals based on the Euclidean distances. Values at branches are AU *p*-values (red) and bootstraps BP-values (green), based on cp-SSR and n-SSR markers.

The dendrogram based on Euclidian distance calculated for the most informative microsatellite (5 chloroplast and 4 nuclear), showed three major clusters (A, B, and C; Figure 7). *M. murex* formed an independent cluster (A − AU = 100%), *M. scutellata* and *M. sativa*, belonging to two different sections, formed a distinct cluster (B) and their relative branches showed a high AU values (95%), which explained that those subdivisions are strongly supported by the data. The third cluster (C—Figure 7), including the large part of the samples, was divided in six subclusters able to well distinguish the *Medicago* species: C1 grouped *M. orbicularis* belonging to section Orbiculares; C2 cluster comprised the specimens belonging to *M. littoralis*, two belonging to *M. minima* and one to *M. polymorpha*; the subcluster C3 incorporated the samples of *M. rugosa* and one of *M. minima*; C4 was identified as the cluster including *M. lupulina* specimens; C5 was formed by the *M. murex*; C6 grouped the samples belonging to *M. arabica*.

#### 3.4.2. Unweighted Pair-Group Method with Arithmetic Averages Using NTSYS-PC Software

**Figure 8:**
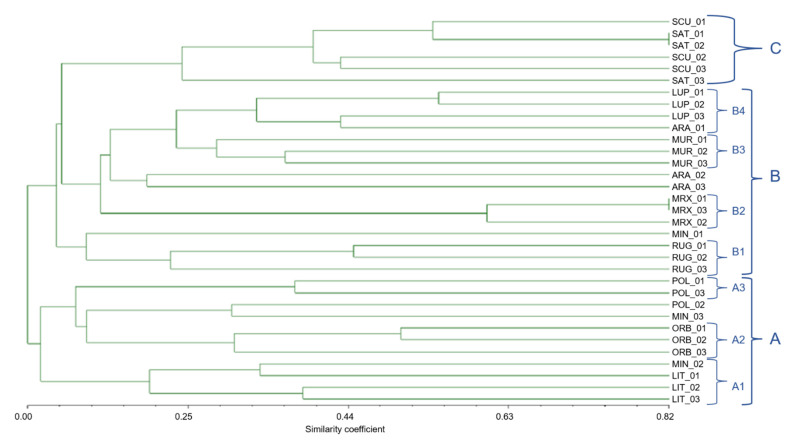
Dendrogram based on Jaccard similarity coefficients, computed from UPGMA, showing relatedness among the 11 *Medicago* species belonging to different sections. Clustering (A, B, and C) is based on both chloroplast and nuclear SSR markers.

The dendrogram derived from Jaccard coefficient (Figure 8) based on similarity matrix of the *Medicago* species showed three major groups (A, B, and C), and the similarity coefficient ranged from 5 to 82%. The cluster A can be further divided into three sub-groups (A1, A2, and A3) having different degrees of similarity; the A1 was represented by *M. littoralis* (subsection Pachyspirae), A2 comprised *M. orbicularis* (section Orbiculares), whilst A3 included 2 of the 3 specimens of *M. polymorpha*. The group B embodied five species of the Spirocarpos section, with four sub-clusters; *M. rugosa* formed a separated subgroup B1; B2 included *M. murex*, whilst B3 and B4 were formed by *M. muricoleptis* and *M. lupulina*, respectively. However, one sample of *M. arabica*, included in cluster B4, showed roughly 35% of similarity with *M. lupulina*.

Finally, this dendrogram highlighted the fact that *M. arabica* specimens had a very high biodiversity; in fact, their Jaccard similarity index was equal to 20% between ARA2 and ARA3. Similarly, the specimens identified as *M. minima* showed a very low similarity index, and were wide spread among all the identified clusters.

The cluster C embodied *M. scutellata* and *M. sativa*, belonging to two different sections, (e.g., Spirocarpos and Medicago, respectively). 

## 4. Discussion

In addition to morphological identification and study of diagnostic traits, molecular characterization can help to solve a number of taxonomic ambiguities [30]. In the present study, we assessed, for the first time, whether nuclear and/or chloroplast microsatellites may be suitable for the differentiation of 11 *Medicago* species (and relative sections) collected in South Italy in Campania region. 

The high level of synteny between legume genomes allowed the transferability of SSR markers developed in *M. truncatula* [33] (more than 500 SSR) to the other *Medicago* species [21,34,35,36]. Contrariwise, chloroplast genomes have lower evolutionary rate, are not recombining predominantly, maternally inherited, highly conserved across genera, and often distributed throughout non-coding regions.

Compared to morphological data, molecular markers can provide highly reliable information, in fact, they are insensitive to environmental variations and, furthermore, not subject to personal interpretation. Previous studies showed that molecular markers are suitable for revealing phylogenetic relationships among different *Medicago* species and also to estimate the genetic diversity. In fact, different molecular markers (IRAP, REMAP, ISSR, SSR, RAPD, and AFLP) have been used to estimate the genetic diversity and evaluate the phylogenetic relatedness in *Medicago* species [32,48,61,62,63,64,65,66,67,68,69,70,71].

A noteworthy result obtained with the present study is that, on a quite limited surveyed area, a significant number of Medicago species were present, including the rare *M. muricoleptis* species, which was recorded in the Campania region for the first time only in 2019 [72]. The species richness in the study area is highly remarkable, regardless the level of anthropic pressure on the area and the intensive land uses (e.g., agricultural, industrial, tourist etc.). However, this apparent paradox is in line with the renewed tolerance to disturbance of many *Medicago* species [73]. 

### 4.1. Intra Species Diversity

Allele number of nuclear SSR within species varied from 3 to 10, with the highest value observed for *M. scutellata*, *M. arabica*, and *M. minima*, and the lowest one for *M. murex*.

For all of the species studied, the results show a low amount of heterozygosity (*H_o_* varied between 0.00 and 0.33, and *H_e_* between 0.11 and 0.44). The highest fixation index was found in *M. muricoleptis* and the lowest one in *M. polymorpha*. The high self-pollination detected in *M. polymorpha* (*H_o_* = 0.00, *H_e_* = 0.11) likely contributed to lower the overall level of the observed heterozygosity. A low heterozygosity was also observed in the case of *M. lupulina*, confirming the data of Yan and co-workers [67], who reported that the *H_o_* within *M. lupulina* populations was 0.017, ranging between 0.00 and 0.04. Nevertheless, the limited heterozygosity cannot be due to outcrossing events, since it was found that the breeding system of *M. lupulina* varies from complete self-pollination to extensive outcrossing and, although honeybees show great interest for *M. lupulina* flowers, under natural conditions, neither pollinating insects nor wind are absolutely necessary for its fertilization [74,75].

### 4.2. Diversity among Species

This study included 11 *Medicago* species (whit a number of globally poorly studied species) and has been the first survey of this type for Campania region and Italy, so that it is not possible to compare with similar study cases or previous national investigations. Although genetic diversity has fundamental importance for species survival [76,77], few studies are available on less common and endangered species, such as the island endemic *M. citrina* and others [64]. In fact, at the global level, genetic studies are mainly concentrated on *M. truncatula, M. polymorpha*, on the *M. sativa–M. falcata* complex [78], and on *M. truncatula*, from which widely used SSR markers were identified, as reported in Diwan et al. [62]. In the same vein, Min et al. [78] found that the mean value of information content of the SSR polymorphisms detected in diverse accession of *M. truncatula*, was as high as 0.71. This number is usually more than 0.70 for annual medics [62,78]. 

Lesins and Lesins [22] and Small and co-workers [24] considered a variety of genetic and morphological traits, such as chromosome number, presence of woody tissue, or cotyledon structure that support recognition of infrageneric taxa and the delimitation of species within the genus *Medicago* [13]. However, results presented by Steele et al. [13] suggest that section Lupularia, containing the two species *M. lupulina* and *M. secundiflora*, should no longer be recognized. The same authors also propose considering a reduced subsection Leptospirae, with *M. lupulina, M. coronata, M. disciformis, M. minima*, and *M. tenoreana*. Interestingly, we detected a relatedness among *M. lupulina* and *M. minima* in the PCoA plot (UPGMA tree was drawn using Maximum Parsimony method). Although they are traditionally included in two different sections, PCoA plot and UPGMA dendrogram clearly highlighted a limited genetic distance between *M. sativa* and *M. scutellata*, which is thought to be a polyploid derivative of a hybrid between a 2n = 16 species and a 2n = 14 [20]. In another global study of the *Medicago* genus [77], although based on isoenzyme banding pattern, among fifty *Medicago* species, representing eight sections (Spirocarpos, Lunatae, Buceras, Medicago, Hymerocarpos, Lupularia, Orbiculares, and Heynianae sections), the dendrograms, based on cluster analysis of isozyme data, showed relatedness among them. Otherwise, population genetics studies on *Medicago* species in Iran have been mainly limited to *M. sativa* [68]. On the basis of the data produced with this study on 11 *Medicago* species assayed through a selection of highly informative nuclear and chloroplast markers, it is possible to conclude that, in most cases and with few exceptions, the distinction of the sections (and subsections) of the genus *Medicago* is supported by the detected genetic diversity. 

### 4.3. Effectiveness of N-SSR and Cp-SSR Markers

Importantly, n-SSR were more effective than cp-SSR markers to separate *Medicago* species from the genetic point of view. Our n-SSR-based clustering of *Medicago* species chiefly agreed with the recent taxonomic classifications [4]. In fact, the species belonging to Spirocarpos section were grouped together in the same clade, and species belonging to Orbiculares section formed a distinct group. Nonetheless, for Medicago section *M. sativa* specimens were grouped in the same clade together with *M. scutellata*, and this could be due to the small number of microsatellites markers used.

Furthermore, n-SSR markers placement of the species based on Bayesian clustering analysis (which sets individuals to groups in relation with genotype), agreed with their placement based on pvclust non-Bayesian clustering approach. An unexpected clustering of *M. polymorpha* specimens was observed, which is not surprising and it resulted from a low level of polymorphism within species and the limited number of DNA SSR-markers used.

At the same time, the microsatellites employed in our study (both chloroplast and nuclear) may provide a useful tool for the management of germplasm repository. In fact, such an assay would be particularly relevant and effective, with a relative low cost and time spent, and for the possibility of automation. Our results confirm, once more, the potentiality and the effectiveness of the SSRs in genetic study and, in particular, it provides a successful experience in *Medicago* species discrimination and suitable for further studies on this genus.

## 5. Conclusions

This study focused on the discrimination power of n-SSR and cp-SSR for 11 *Medicago* species collected in a very limited area, and including M. muricoleptis, a species recorded for the first time in 2019 in Campania region. Nuclear microsatellites have proven very effective for species discrimination. Otherwise, cp-SSR resulted informative as well, being related to the common phylogenetic origin of *Medicago* species. Therefore, this innovative approach, which combined data from both n-SSR and cp-SSR, has been proven highly informative and allowed to distinguish *Medicago* species in relation to their common phylogenetic origin. 

## Figures and Tables

**Table 1:** Scientific names, sections, and subsections of the 11 *Medicago* species collected in Campania, with geographical coordinates of the collection sites (WGS84) [4]. The status in Campania follows the checklist of the Italian flora (2021).

Species	Species Code	Status in Campania	Section	Subsection	Latitude Longitude
*Medicago arabica* (L.) Huds.	ARA	Native	*Spirocarpos* Ser.	*Spirocarpos*	40.774547 14.788292
*Medicago littoralis* Rohde ex Loisel.	LIT	Native	*Spirocarpos* Ser.	*Pachyspirae* (Urb.) Heyn	40.069056 15.513661
*Medicago lupulina* L.	LUP	Native	*Spirocarpos* Ser.	*Lupularia* (Ser. in DC.) E.Small	40.772229 14.789310
*Medicago minima* (L.)	MIN	Native	*Spirocarpos* Ser.	*Spirocarpos*	40.617235 14.993029
*Medicago murex* Willd.	MRX	Native	*Spirocarpos* Ser.	*Pachyspirae* (Urb.) Heyn	40.140534 15.557399
*Medicago muricoleptis* Tineo	MUR	Native	*Spirocarpos* Ser.	*Intertextae* (Urb.) Heyn	40.071391 15.503251
*Medicago orbicularis* (L.) Bartal.	ORB	Native	*Orbiculares* Urb.	-	40.539465 15.116915
*Medicago polymorpha* L.	POL	Native	*Spirocarpos* Ser.	*Spirocarpos*	40.071492 15.511916
*Medicago rugosa* Desr.	RUG	Native	*Spirocarpos* Ser.	*Rotatae* (Urb.) Heyn	40.554012 14.981541
*Medicago sativa* L.	SAT	Archaeophyte	*Medicago* L.	*Medicago*	40.774438 14.788476
*Medicago scutellata* (L.) Mill.	SCU	Native	*Spirocarpos* Ser.	*Rotatae* (Urb.) Heyn	40.541022 15.114211

**Table 2:** Diversity statistics of informative nuclear and chloroplast microsatellites used to study the 11 *Medicago* species.

Marker	Locus	An	PIC	He	Fis	*F*_ST_	Nm	PR
**n-SSRs**	MTIC503	11	0.847	0.861	0.813	0.625	0.150	4.0
	MTIC559	8	0.772	0.795	0.158	0.652	0.133	3.0
	MTIC563	9	0.672	0.695	0.467	0.674	0.121	6.0
	MTIC564	12	0.827	0.844	0.360	0.549	0.205	5.0
	Mean	10	0.780	0.799	0.449	0.625	0.152	4.5
**cp-SSRs**	CCMP2	12	0.685	0.878				
	CCMP4	10	0.802	0.821				
	CCMP6	7	0.644	0.694				
	CCMP7	10	0.860	0.873				
	CCMP10	17	0.891	0.899				
	Mean	11	0.776	0.833				

An, alleles number; PIC, polymorphism information content; He, Mean Nei’s gene diversity. Fis, inbreeding index; *F*_ST_, fixation index; Nm, number of migrants; PR, private alleles.

**Table 3:** Genetic diversity among the 11 *Medicago* species investigated in this study.

Marker	Species	N	Na	Ne	I	Ho	He	F	Nm	A
**n-SSRs**	SCU	3	2.3	2.1	0.6	0.17	0.4	0.69	0.19	7
	SAT	3	2.3	1.9	0.7	0.33	0.44	0.29	0.4	8
	LUP	3	1.5	1.2	0.2	0.17	0.12	−0.33	−0.64	6
	ARA	3	2.2	1.9	0.7	0.25	0.46	0.32	0.35	8
	MUR	3	1.7	1.6	0.4	0.08	0.26	0.73	0.23	5
	POL	3	1.3	1.2	0.16	0	0.11	1	0.22	3
	ORB	3	2	2	0.55	0.17	0.33	0.5	0.3	5
	MIN	3	2	1.7	0.47	0.17	0.28	0.5	0.33	6
	LIT	3	2	1.6	0.53	0.17	0.35	0.56	0.26	8
	RUG	3	1.5	1.3	0.27	0.08	0.18	0.4	0.49	5
	MRX	3	2	1.9	0.6	0.25	0.4	0.33	0.39	8
	Mean	3	1.9	1.7	0.48	0.17	0.3	0.45	0.23	6.3
	SE	0	0.1	0.1	0.06	0.04	0.04	0.1	0.09	0.5
**cp-SSRs**	SCU	3	2.2	2	0.73		0.49			10
	SAT	3	2	1.9	0.6		0.4			8
	LUP	3	2.2	2.1	0.69		0.44			9
	ARA	3	2.2	2	0.73		0.49			10
	MUR	3	2.4	2.3	0.82		0.53			8
	POL	3	2.4	2.3	0.82		0.53			8
	ORB	3	2.2	2.1	0.69		0.44			9
	MIN	3	2.6	2.5	0.91		0.58			10
	LIT	3	2.4	2.3	0.82		0.53			7
	RUG	3	2.4	2.4	0.79		0.49			8
	MRX	3	1.2	1.2	0.13		0.09			5
	Mean	3	2.1	2	0.67		0.44			8.4
	SE	0	0.1	0.1	0.05		0.03			0.4

N, number of individuals analyzed; Ne, number of effective alleles per locus; No, Number of alleles per locus; A, total number of alleles or haplotypes detected for 11 species in n-SSRs andcp-SSRs; I, Shannons diversity index; He, gene diversity; Ho, observed heterozygosity; Nm, number of migrants; F, inbreeding index.

**Table 4:** Analysis of genetic differentiation between the 11 *Medicago* species by AMOVA with cp-SSR and n-SSR markers. df, degrees of freedom; MS, Mean squares; *F*_ST_, fixation index.

Marker	Source of Variation	Df	Sum of Squares	MS	Variance Component	Variation (%)	*F*_ST_ (*p*-Value)
**n-SSR**							
	Among species	10	66.288	6.629	0.884	52	0.01
	Among individuals within species	22	29.167	1.326	0.496	29	
	Within Individuals	33	11.000	0.333	0.333	19	
	Total	65	106.455		1.713	100	
**cp-SSR**							
	Among species	10	182,589	18,258	932	6	0.028
	Within species	22	340,128	15,460	15,460	94	
	Total	32	522,717		16,393	100	

## Data Availability

Included in the article or Supplementary Material.

## Supplementary Materials

The following are available online at https://www.mdpi.com/article/10.3390/genes13010097/s1, Table S1: Molecular and genetic information on the four nuclear microsatellites loci used in the study; Table S2: Molecular and genetic information on the five chloroplast microsatellites used in the study; Table S3: *F*_ST_ values among species.

## Abbreviations

AMOVA Analysis of molecular variance; cp-SSRs, chloroplast microsatellites markers; n-SSRs, nuclear microsatellites markers; PCoA, principal coordinates analysis; PIC, Polymorphism information content; SSR, simple sequence repeat; UPGMA, unweighted pair-group method with arithmetic averages.
