# Supplementary material for: Genetic Diversity and Differentiation of Eleven Medicago Species from Campania Region Revealed by Nuclear and Chloroplast Microsatellites Markers
Source: Genes (Basel). 2021 Dec 31;13(1):97. doi: 10.3390/genes13010097 (PMC8774365; doi:10.3390/genes13010097)
Supplement: Supplementary file 1 [file genes-13-00097-s001.zip › Table S1.pdf]

Table S1. Molecular and genetic information on the four nuclear microsatellites loci used in the study.

Locus code	Primer sequences 5′ 3′	Core sequence	Linkage group	Origin	Reference
MTIC503 (=TPG20C) (=FMT08)	CAATCACTGGAAGCAAGGT AGCCTGCTCATTTGTATTGC	$[CT]_{16}$, $[CA]_{7}$	7	A	[48,35]
MTIC559	GGGTTTTTGATCCAGATCTT AAGGTGGTCATACGAGCTCC	$[TTC]_{8}$	4	B	[31]
MTIC563	AATTCCTTCGGCATCAACAC TGCCGAAATTGTTAGTTTGG	$[CAA]_{8}$	1	A	[46]
MTIC564	GCCGATGGTACTAATGTAGG AAATCTTGCTTGCTTCTCAG	$[GA]_{13}$	2	C	[32]

Primer sequences: (first line: left primer, second line: right primer); Origin: (A): Microsatellite-enriched genomic library; (B): Expressed Sequence Tags (ESTs); (C): Bacterial Artificial Chromosome (BAC).
