# Supplementary material for: Genetic Diversity and Differentiation of Eleven Medicago Species from Campania Region Revealed by Nuclear and Chloroplast Microsatellites Markers
Source: Genes (Basel). 2021 Dec 31;13(1):97. doi: 10.3390/genes13010097 (PMC8774365; doi:10.3390/genes13010097)
Supplement: Supplementary file 1 [file genes-13-00097-s001.zip › Table S2.pdf]

Table S2. Molecular and genetic information on the five chloroplast microsatellites used in the study.

Code	Primer Sequences 5′ 3′	Position (pb)	Location	SSR motif	size in (*Melilotus parviflora*) (pb)	Reference
CCMP2	GATCCCGGACGTAATCCTG<br>ATCGTACCGAGGGTTCGAAT	8609	5′ to trnS	(A)11	70	Weising and Gardner 1999 [47]
CCMP4	AATGCTGAATCGAYGACCTA<br>CCAAAATATTBGGAGGACTCT	12872	atpF intron	(T)13	130	
CCMP6	CGATGCATATGTAGAAAGCC<br>CATTACGTGCGACTATCTCC	45119	ORF 77–ORF 82 intergenic	(T)5C(T)17	120	
CCMP7	CAACATATACCACTGTCAAG<br>ACATCATTATTGTATACTCTTTC	57339	atpB–rbcL intergenic	(A)13	160	
CCMP10	TTTTTTTTTAGTGAACGTGTCA<br>TTCGTCGDCGTAGTAAATAG	86694	rpl2–rps19 intergenic	(T)14	190	

Degenerate positions : Y (= C or T), B (= G, C, or T) and D (= A, T, or G).
