# Supplementary material for: Genetic Diversity and Differentiation of Eleven Medicago Species from Campania Region Revealed by Nuclear and Chloroplast Microsatellites Markers
Source: Genes (Basel). 2021 Dec 31;13(1):97. doi: 10.3390/genes13010097 (PMC8774365; doi:10.3390/genes13010097)
Supplement: Supplementary file 1 [file genes-13-00097-s001.zip › Table S3.pdf]

Table S3. F_{st} values among species.

SCU	SAT	LUP	ARA	MUR	POL	ORB	MIN	LIT	RUG	MRX	
0											SCU
0.091	0										SAT
0.176	0.128	0									LUP
0.286	0.19	0.061	0								ARA
0.2	0.2	0.057	0.04	0							MUR
0.333	0.333	0.263	0.245	0.154	0						POL
0.241	0.241	0.091	0.077	0.071	0.2	0					ORB
0.154	0.154	0.02	0.077	0.037	0.2	0.071	0				MIN
0.22	0.22	0.103	0.091	0.069	0.179	0.179	0.085	0			LIT
0.2	0.2	0.057	0.04	0	0.154	0.154	0.037	0.069	0		RUG
0.333	0.333	0.176	0.2	0.2	0.333	0.043	0.154	0.08	0.2	0	MRX
